# A Prognostic Model for Development of Profound Shock among Children Presenting with Dengue Shock Syndrome

**DOI:** 10.1371/journal.pone.0126134

**Published:** 2015-05-06

**Authors:** Phung Khanh Lam, Dong Thi Hoai Tam, Nguyen Minh Dung, Nguyen Thi Hanh Tien, Nguyen Tan Thanh Kieu, Cameron Simmons, Jeremy Farrar, Bridget Wills, Marcel Wolbers

**Affiliations:** 1 Oxford University Clinical Research Unit, Hospital for Tropical Diseases, Ho Chi Minh City, Viet Nam; 2 University of Medicine and Pharmacy of Ho Chi Minh City, Ho Chi Minh City, Viet Nam; 3 Hospital for Tropical Diseases, Ho Chi Minh City, Viet Nam; 4 Centre for Tropical Medicine, Nuffield Department of Clinical Medicine, University of Oxford, Oxford, United Kingdom; Institute of Tropical Medicine (NEKKEN), Nagasaki University, JAPAN

## Abstract

**Purpose:**

To identify risk factors and develop a prediction model for the development of profound and recurrent shock amongst children presenting with dengue shock syndrome (DSS)

**Methods:**

We analyzed data from a prospective cohort of children with DSS recruited at the Paediatric Intensive Care Unit of the Hospital for Tropical Disease in Ho Chi Minh City, Vietnam. The primary endpoint was “profound DSS”, defined as ≥2 recurrent shock episodes (for subjects presenting in compensated shock), or ≥1 recurrent shock episodes (for subjects presenting initially with decompensated/hypotensive shock), and/or requirement for inotropic support. Recurrent shock was evaluated as a secondary endpoint. Risk factors were pre-defined clinical and laboratory variables collected at the time of presentation with shock. Prognostic model development was based on logistic regression and compared to several alternative approaches.

**Results:**

The analysis population included 1207 children of whom 222 (18%) progressed to “profound DSS” and 433 (36%) had recurrent shock. Independent risk factors for both endpoints included younger age, earlier presentation, higher pulse rate, higher temperature, higher haematocrit and, for females, worse hemodynamic status at presentation. The final prognostic model for “profound DSS” showed acceptable discrimination (AUC=0.69 for internal validation) and calibration and is presented as a simple score-chart.

**Conclusions:**

Several risk factors for development of profound or recurrent shock among children presenting with DSS were identified. The score-chart derived from the prognostic models should improve triage and management of children presenting with DSS in dengue-endemic areas.

## Introduction

Dengue is a common systemic viral infection that generates a considerable economic burden for healthcare systems in tropical and subtropical regions of the world, with an estimated 40% of the world’s population living in areas of risk. [1,2] The clinical manifestations range from self-limited febrile illness, to severe disease including dengue shock syndrome (DSS), a form of hypovolaemic shock resulting from excessive plasma leakage due to a transient increase in systemic vascular permeability. DSS is a potentially fatal disease with reported mortalities varying from <1% to 10% depending on the patient mix encountered, the clinical facilities available, and the experience of the local healthcare personnel. [3–6]

There is no specific therapy for dengue and effective case-management relies on good supportive care, in particular judicious use of parenteral fluid therapy to counteract plasma leakage. The severity of leakage in patients with DSS ranges from relatively mild, with a good response to prompt initial volume resuscitation, through to profound plasma leakage requiring large volumes of parenteral fluid therapy together with sophisticated intensive-care management of bleeding and the other complications that may accompany severe shock. In such cases, fluid overload is a significant contributor to morbidity and mortality, and balancing parenteral fluid therapy at a level just sufficient to maintain cardiovascular stability and critical organ perfusion during the phase of vascular leakage requires considerable skill and experience. In hyperendemic regions, where many DSS cases may present each day, identification of patients with profound leakage early in the course of shock could facilitate more effective use of limited resources.

Prognostic models for severe disease can enhance a physician’s clinical decision-making processes. [7] Several prognostic models (PRISM, PIM, PELOD) have been developed to characterise children with severe illness admitted to western paediatric intensive care units (PICUs), [8–10] and some analyses suggest that PRISM and PELOD may be useful in identifying DSS patients at risk of dying. [11,12] However, these scoring systems require detailed clinical and laboratory data that are not readily available in most countries where dengue is endemic. Although a number of models have examined risk factors for development of shock among individuals with dengue, [13,14] to date only a single report describes a prognostic model for poor outcome in patients with established DSS. [15] In this study, we analyzed data from a much larger cohort of children (n = 1207) presenting with DSS to a single hospital, aiming to identify risk factors for profound shock, and to develop a robust prognostic model to assist physicians in identifying children likely to require intensive supportive therapy.

## Materials and Methods

### Ethics statement

We utilised data from a large prospective study of children presenting with DSS to the PICU at the Hospital for Tropical Diseases (HTD) in Ho Chi Minh City. The study was approved by the HTD Ethical Committee and the Oxford Tropical Research Ethics Committee. For each participant written informed consent was given by a parent or guardian.

### Study procedures and management protocols

Details regarding the design of this study, and a description of participant characteristics, clinical evolution and management have been described previously. [16] Briefly, children less than 15 years old admitted to PICU at HTD with established DSS were eligible for enrolment. We collected demographic information, history and examination findings within 2 hours of onset of shock, and subsequently recorded detailed information on fluid usage and clinical outcomes daily during the hospitalization. Specimens for dengue diagnostics were collected at enrolment and discharge, and diagnosis was confirmed with dengue IgM and IgG capture ELISAs and/or RT-PCR as previously described. [16]

Patients were managed according to HTDs protocol-driven guidelines for paediatric DSS by the same core group of experienced physicians throughout the study period. In subjects presenting with compensated shock (i.e. with pulse pressure [PP] narrowing to ≤20 mm Hg but systolic pressure maintained in the normal range for age, and with signs of impaired perfusion) the guidelines specify a standard regimen of 25 ml/kg Ringer’s lactate solution over 2 hours, with colloid solutions (dextran or starch depending on availability) reserved for more severe cases presenting with decompensated/hypotensive shock or without a detectable output. Subsequently, all patients receive a standardized schedule of Ringer’s lactate, reducing the rate of fluid perfusion at fixed time-intervals to 3 ml/kg/hr over 8 hours.

Recurrent shock is commonly observed during the critical period for leakage, with some patients experiencing multiple episodes over 24–48 hours until the re-absorptive phase of the illness commences. [17] Recurrent shock was diagnosed when the PP narrowed again to ≤20mmHg, in association with tachycardia, cool extremities, and a rising haematocrit, and was managed with 10–15 mL/kg infusions of colloid over 30–60 minutes, with other supportive therapy given according to the clinical scenario. Since all DSS patients have significant coagulopathy and thrombocytopenia central venous pressure (CVP) monitoring is considered high-risk; HTD’s guidelines set the threshold to proceed to CVP guided fluid management as the need for more than two colloid boluses. Inotropic support is recommended for patients with persistently unstable haemodynamic indices despite volume resuscitation; occasionally these agents are used after a single bolus of colloid if there are particular concerns about the potential for adverse effects of additional colloid therapy (severe coagulopathy, early evidence of fluid overload, renal compromise). The guidelines for blood product usage are conservative, with whole blood reserved for frank haemorrhage causing circulatory compromise, or, rarely, for rescue therapy in patients with prolonged unresponsive shock without overt evidence of bleeding.

### Clinical outcomes and candidate predictors

We assessed two outcomes in this study. First, “*recurrent shock*” defined as at least one episode after the initial resuscitation, and second “*profound DSS*”, defined as either a) requirement for two or more colloid boluses (either crystalloid resuscitation for compensated shock at presentation plus two or more episodes of recurrent shock, or colloid resuscitation for decompensated shock at presentation plus one or more episodes of recurrent shock) or b) requirement for inotropes in addition to colloid therapy, to maintain cardiovascular stability. Although major bleeding may occur as a consequence of profound or prolonged shock it is rarely a primary contributor to DSS in children. However, to limit potential confounding, patients in whom major bleeding was diagnosed prior to fulfilment of the criteria for profound shock were excluded from the analysis.

For a number of reasons we chose profound DSS as the primary outcome of interest for development of the final models. Differences in initial resuscitation between patients with compensated or decompensated shock (use of crystalloid versus colloid fluids) may have influenced the likelihood of developing recurrent shock subsequently. Secondly a large proportion of cases experiencing their first episode of recurrent shock recover fully following a single colloid bolus without requiring additional supportive therapy. This composite outcome measure also reflects the local threshold for concern regarding severe disease, as indicated by the recommendation to proceed to CVP monitoring in cases requiring more than two colloid boluses.

Pre-defined candidate predictors, all assessed within 2 hours of onset of shock, are described in Table A in S1 File and were chosen based on clinical experience and evidence from the published literature. [18–20] As PP and systolic blood pressure (SBP) are closely linked and some patients may present with no detectable blood pressure, we defined a hemodynamic index to allow all patients to be classified into one of three ordered categories representing their initial cardiovascular status. The hemodynamic index is 1 when PP≥10 mmHg and SBP is maintained above the lower limit of normal for age (i.e. ≥80 mmHg if under 5 years, or ≥90 mmHg if aged 5 years or more), 2 when PP<10 mmHg or SBP is below the lower limit for age, and 3 if the blood pressure is unmeasureable.

### Statistical analysis

The study population included all patients enrolled between 2003 and 2009 in the prospective observational study of paediatric DSS previously described, [16] for whom there was a confirmed virological/serological diagnosis of dengue. Logistic regression was the main statistical model for univariate and multivariable analyses. The multivariable logistic model included all pre-defined candidate predictors, with continuous predictors modelled as linear terms. A detailed assessment of model assumptions was performed and if there was clear evidence of non-linearity or interactions between predictor variables, we added corresponding terms to the final full model. We then simplified this model using stepwise backwards model selection based on the Akaike Information Criterion (AIC). For the primary outcome of profound DSS, we additionally compared the performance (area under the ROC curve (AUC), calibration-in-the-large and calibration slope [21]) of the logistic regression models to several alternative statistical approaches: logistic regression with variable selection and shrinkage based on the adaptive lasso, classification and regression trees (CART), generalized additive models (GAM), and gradient boosting with trees as base learners. [22] The entire model development process, except for the assessment of model assumptions, was validated using both temporal and internal validation. [21,23] For temporal validation, the models were developed using data from the 939 patients enrolled before 2009 and validated on the 268 patients enrolled during 2009. For internal validation, we obtained model performance using repeated 10-fold cross-validation. Additional details regarding the prognostic model development are presented in the S1 File. The model that showed the most favourable trade-off between performance and simplicity was chosen as the basis for a score chart. [24] We also assessed the performance of the prognostic model for recurrent shock developed by Huy et al using our dataset. [15]

For multivariable models, missing covariate values (<2% for all candidate predictors) were imputed with the median of non-missing values for continuous variables, or the most frequent category for categorical variables. Univariate analyses were based on complete case analyses. All analyses were performed with the statistical software R version 3.1.2 and its companion packages. [25–30]

## Results

**Fig 1:**
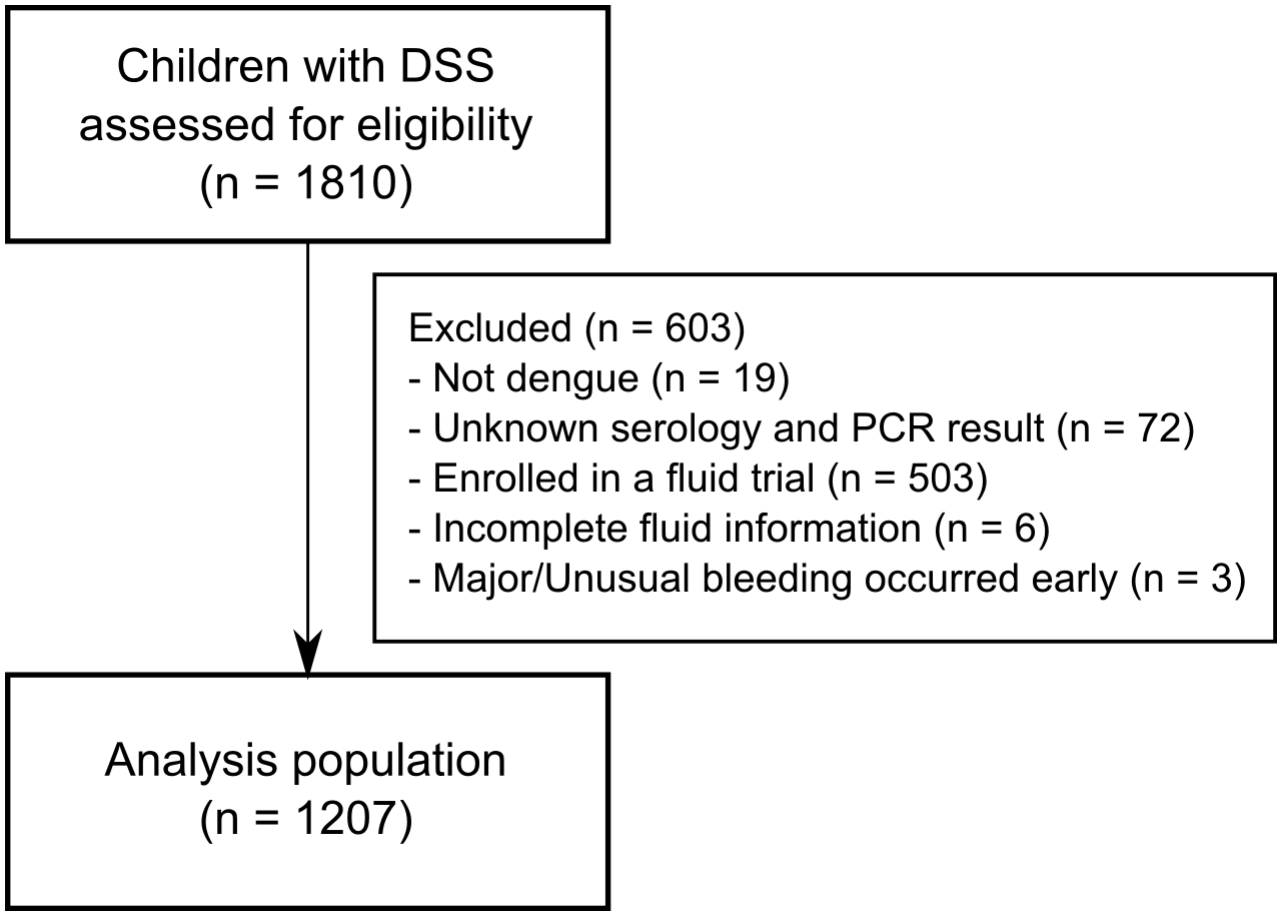
The study flow-chart.

**Fig 2:**
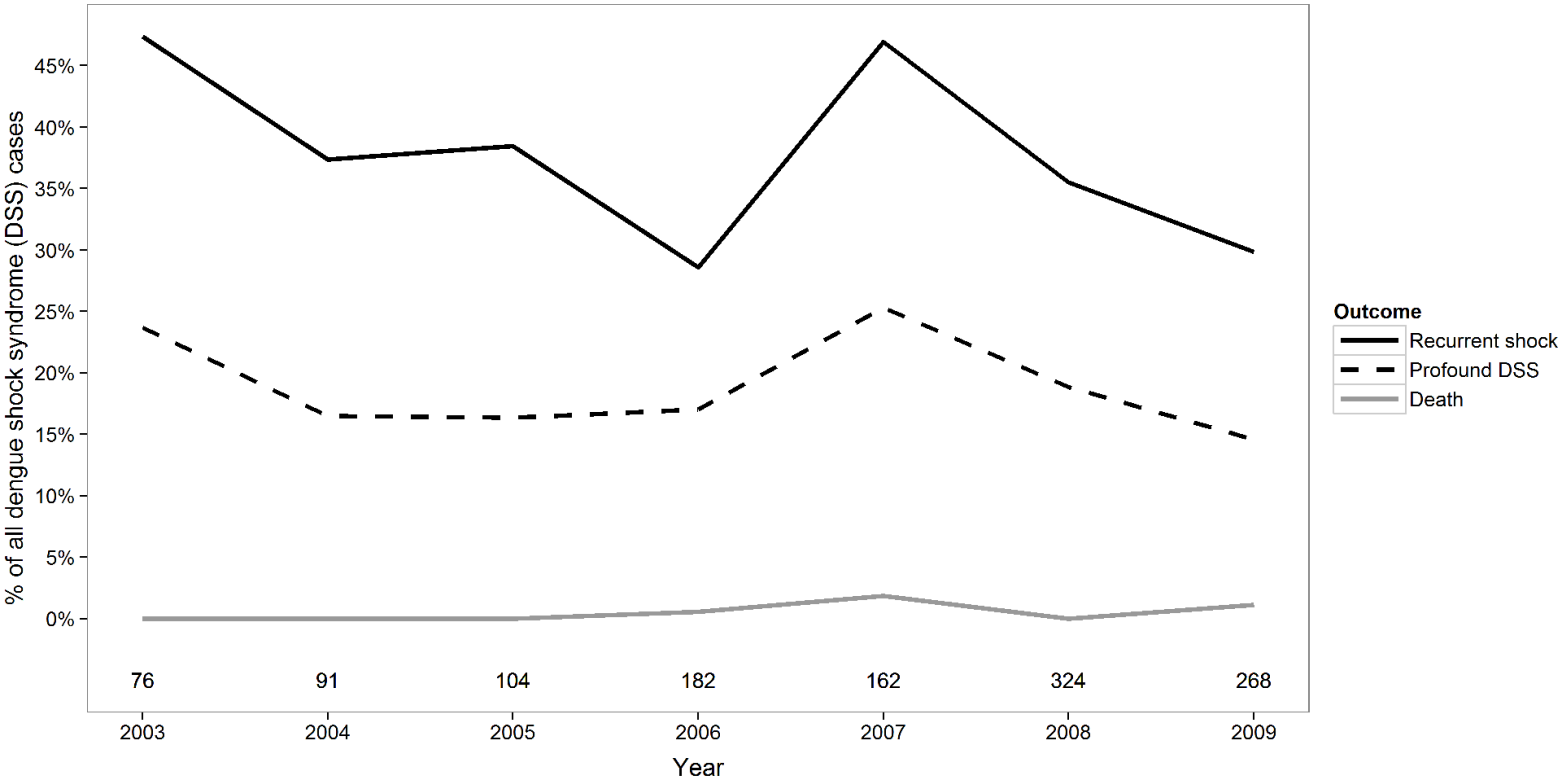
Frequency of adverse clinical outcomes over time for the patients enrolled in the study (N = 1207). The numbers shown below the line graphs indicate the total number of DSS cases enrolled in the study each year.

A total of 1216 children with DSS were enrolled in the observational study during the relevant time-period and confirmed to have dengue. Six children, for whom insufficient information regarding fluid usage was available, and three children, in whom major/unusual bleeding occurred early in the illness course precluding assessment of the primary outcome, were excluded from the analysis (Fig 1). In total, 433/1207 (36%) of the children had at least one episode of recurrent shock and 222/1207 (18%) developed profound DSS (Table 1). Fifty-four children required inotropic support; in 50 cases inotropes were commenced after two boluses of colloid, while in the remaining four cases inotropic support was started earlier, in preference to additional colloid therapy, as the treating clinicians were concerned by significant bruising and/or early clinical signs of fluid overload. Seventeen children went on to develop major mucosal bleeding, and 6 of these cases (two of whom also displayed multi-organ failure) subsequently died. No systematic time trends were observed for the prevalence of profound DSS over the study period but we observed a small and borderline significant decline in the prevalence of recurrent shock (linear trend test: p = 0.03) (Fig 2).

**Table 1:** Baseline characteristics and outcomes of study participants (N = 1207).

Characteristics	n	Summary statistics	
**Demographic characteristics**			
Age [years]	1207	10	(7–12)
Gender: male	1207	645	(53)
**Clinical features at presentation with shock**			
Weight [kg]	1207	29	(21–38)
Day of illness	1207	5	(5–6)
Pulse pressure [mmHg]	1207	20	(15–20)
Systolic blood pressure [mmHg]	1207	90	(85–100)
Pulse rate [beats/min]	1207	120	(110–140)
Hemodynamic Index	1207		
Group 1		829	(69)
Group 2		300	(25)
Group 3		78	(6)
Temperature ≥ 38°C	1206	108	(9)
Hemorrhage	1207		
None		398	(33)
Skin only ^a^		769	(64)
Mucosal ^b^		40	(3)
Abdominal tenderness	1205	787	(65)
Liver size [cm]	1195	2	(1–2)
Ascites/Pleural effusion ^c^	1204	12	(1)
Haematocrit [%]	1186	50	(47–52)
Platelet count [cells/mm^3^]	1188	38000	(26000–54000)
AST [IU/L]	910	133	(89–218)
Serotype	1167		
DENV-1		658	(56)
DENV-2		281	(24)
DENV-3		19	(2)
DENV-4		7	(1)
Mixed		8	(1)
Negative		194	(17)
**Outcomes**			
Recurrent shock	1207	433	(36)
Profound DSS ^d^	1207	222	(18)
Inotropic support	1207	54	(4)
Severe bleeding	1207	17	(1)
Organ failure	1207	2	(<1)
Death	1207	7	(1)

Summary statistics are median (interquartile range) for continuous variables and frequency (percentage) for categorical variables. n refers to the number of subjects with non-missing values.

AST: aspartate aminotransferase

DSS: dengue shock syndrome

^a^ including petechiae (775/1207), bruising and purpura (39/1207)

^b^ including epistaxis/gum bleeding (17/1207) and gastrointestinal/vaginal bleeding (26/1207)

^c^ detected by clinical assessment

^d^ includes 89 patients presenting with hypotensive/decompensated shock who had ≥1 recurrent shock episode, 129 patients presenting with compensated shock who had ≥2 recurrent shock episodes, and 4 patients treated with inotropic drugs after their first episode of recurrent shock.

Apart from platelet count, gender, bleeding, abdominal tenderness, and liver size, all pre-defined candidate predictors showed a significant association with both outcomes in the univariate analysis (Table D in S1 File). Female gender was significantly associated with an increased risk of profound DSS (p = 0.04) but not with the risk of recurrent shock (p = 0.85). Younger age, earlier day of illness at shock, higher temperature, faster pulse rate, higher haematocrit and worse hemodynamic status in females, were all independently associated with both profound DSS and recurrent shock in the multivariable logistic models (Table 2). There was no convincing evidence that any of the continuous predictors affected the outcomes non-linearly. However, the effect of hemodynamic status on both outcomes was significantly stronger in females (Table C in S1 File) and we thus included a gender-hemodynamic status interaction in the multivariable models.

**Table 2:** Adjusted effects of candidate predictors on clinical outcomes (full logistic model and reduced model with variable selection) in all study participants (N = 1207).

	Profound DSS		Recurrent shock	
	Full model	Reduced model	Full model	Reduced model
**Candidate predictors**				
Age [+1 year]	0.86 (0.80, 0.93)	0.87 (0.83, 0.92)	0.90 (0.85, 0.96)	0.90 (0.86, 0.94)
Weight [+5 kgs]	1.03 (0.93, 1.14)	-	1.00 (0.92, 1.08)	-
Day of illness [+1 day]	0.79 (0.65, 0.94)	0.78 (0.65, 0.94)	0.78 (0.68, 0.91)	0.79 (0.68, 0.91)
Pulse rate [+10 beats/min]	1.08 (1.03, 1.13)	1.07 (1.03, 1.13)	1.07 (1.02, 1.11)	1.07 (1.02, 1.11)
Temperature [+1°C]	1.58 (1.12, 2.21)	1.59 (1.12, 2.20)	1.85 (1.39, 2.48)	1.85 (1.39, 2.48)
Hemorrhage		-		-
None	1.00 (reference)	-	1.00 (reference)	-
Skin only	0.94 (0.67, 1.33)	-	0.96 (0.73, 1.26)	-
Mucosal	1.22 (0.47, 2.89)	-	1.02 (0.47, 2.14)	-
Abdominal tenderness	0.98 (0.68, 1.42)	-	1.05 (0.78, 1.41)	-
Liver size [+1 cm]	0.96 (0.80, 1.16)	-	1.05 (0.91, 1.22)	-
Haematocrit [+1%]	1.07 (1.03, 1.12)	1.07 (1.03, 1.12)	1.07 (1.03, 1.11)	1.07 (1.03, 1.10)
Platelet count [+10,000 cells/mm^3^]	1.02 (0.98, 1.06)	-	1.00 (0.96, 1.03)	-
Gender: male	1.14 (0.75, 1.73)	1.17 (0.78, 1.77)	1.42 (1.04, 1.95)	1.42 (1.04, 1.93)
Hemodynamic Index—males				
Group 1	1.00 (reference)	1.00 (reference)	1.00 (reference)	1.00 (reference)
Group 2 vs. group 1	0.82 (0.46, 1.41)	0.79 (0.45, 1.36)	0.77 (0.50, 1.16)	0.77 (0.51, 1.16)
Group 3 vs. group 1	1.60 (0.70, 3.61)	1.55 (0.67, 3.49)	0.65 (0.30, 1.41)	0.66 (0.30, 1.43)
Hemodynamic Index—females				
Group 1	1.00 (reference)	1.00 (reference)	1.00 (reference)	1.00 (reference)
Group 2 vs. group 1	2.57 (1.59, 4.15)	2.55 (1.58, 4.12)	1.86 (1.24, 2.79)	1.86 (1.24, 2.79)
Group 3 vs. group 1	3.01 (1.43, 6.36)	3.06 (1.46, 6.45)	1.51 (0.75, 3.05)	1.50 (0.75, 3.03)
**Performance: internal (temporal) validation**				
AUC	0.69 (0.73)	0.69 (0.74)	0.64 (0.69)	0.65 (0.71)
Calibration-in-the-large	-0.02 (-0.49)	-0.02 (-0.50)	-0.003 (-1.47)	-0.004 (-1.47)
Calibration slope	0.87 (1.13)	0.92 (1.22)	0.86 (1.20)	0.93 (1.34)

Performance of the associated prediction models (based on repeated ten-fold cross-validation [internal validation] and temporal validation) is shown at the bottom of the table.

DSS: dengue shock syndrome

Effects are summarized as odds ratio (95% confidence interval).

Interaction tests between gender and hemodynamic index were significant for recurrent shock (p = 0.01) and profound DSS (p = 0.01)

All performance measures were corrected for optimism using 10-times 10-fold cross-validations.

The simplified models for profound and recurrent shock based on stepwise variable selection included the same variables as described above and performed equally well as the full models (Table 2). Discrimination was acceptable with AUC’s of at least 0.69 and 0.65 for profound and recurrent shock, respectively. Calibration-in-the-large in internal validation was close to zero for all models indicating good agreement between the average predicted risks and the observed risks (Table 2). However, in temporal validation, all models developed using data from patients enrolled before 2009 tended to overestimate the risk in 2009 when an unusually low prevalence of severe outcomes was observed (Table 2, Fig 2).

**Fig 3:**
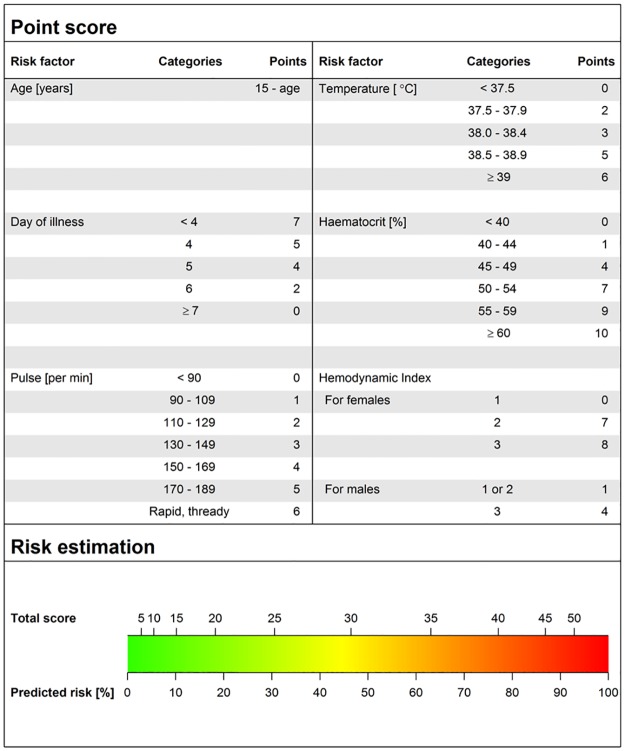
Score-chart for prediction of profound shock. The upper panel assigns a point score for each risk factor while the lower panel assigns the predicted risk of developing profound shock based on the total point sum for all risk factors.

More sophisticated models including adaptive lasso, GAM, CART and boosting did not show an improved performance compared to the simplified logistic model for profound DSS (Table E in S1 File). This model was thus used as the basis for a clinical score chart which assigns points to each predictor value and then translates the total point sum to a predicted risk of a severe outcome (Fig 3, and Figs C and D in S1 File). For example, the total point sum for a 10-year-old girl who presents on day 6 of illness with a pulse rate of 100 beats/min, a temperature of 37.5°C, a haematocrit of 44% and a hemodynamic index of 1 is 11 ({5} + {2} + {1} + {2} + {1} + {0}) which corresponds to a risk of <10% to develop profound shock during hospitalization. Based on this low estimated risk and depending on the available resources and clinical expertise, the treating physician could decide to keep this patient in his/her health facility rather than refer to a higher-level hospital.

We used our dataset to externally assess the performance of the only other published model designed to predict recurrent shock in DSS cases, which is based on five variables—day of illness at hospital admission, plus purpura/ecchymosis, ascites/pleural effusion, platelet count, and pulse pressure, assessed at shock. The model equation yielded an AUC of 0.54 (95% CI: 0.50–0.57) and substantially under-estimated the risk of recurrent shock (average predicted risk 15% compared to an observed risk of 36%).

## Discussion

This study evaluated risk factors for poor outcomes in children with DSS. Younger age, earlier day of illness, higher temperature, faster pulse rate, higher haematocrit, and a worse hemodynamic status (in females) at onset of shock were associated with a higher risk of developing both profound DSS, the primary outcome of this study, and recurrent shock, the secondary outcome. We also developed a robust prediction model for profound shock and present it as a simple score-chart designed to assist decision-making in clinical practice.

The pathognomonic feature of the vasculopathy associated with severe dengue is increased vascular permeability resulting in a transient capillary leak syndrome. Cardiovascular decompensation occurs when plasma losses exceed the capacity for upregulation of the normal compensatory mechanisms that maintain plasma volume within well-circumscribed limits. [31] Several studies have demonstrated a greater risk for vascular leakage and development of DSS among children compared to adults, [6,32–34] probably related to higher intrinsic permeability with younger age, [35] and a relationship with severity of shock is therefore to be expected. Similarly, earlier presentation with DSS implies more severe capillary leakage that quickly overwhelms the capacity for compensation, and this is consistent with the associations demonstrated between profound shock and other markers of leakage severity such as higher haematocrit and more severe hemodynamic compromise at presentation. Higher temperature at onset of shock was also associated with both clinical outcomes; 9% of cases had a temperature of 38 degrees or more at presentation irrespective of the day of illness, [16] possibly indicating a greater viral burden or a more intense immune response in these cases. Several of these factors, and others such as AST identified in the univariate analysis, have also been identified as risk factors for development of shock and/or for more severe dengue disease generally. [6,13,18]

Interestingly, despite the well-established association between vascular leakage and thrombocytopenia in dengue cases in general, we did not find a significant relationship between platelet count and either of the clinical outcomes. [36,37] However, it is probable that the profound thrombocytopenia already present at the onset of shock masked any additional effects. Similarly the absence of any relationship between abdominal tenderness or liver size and shock severity likely reflects the fact that these parameters are closely linked with development of shock per se. Female gender has been identified previously as an independent predictor of mortality in children with DSS, possibly reflecting higher intrinsic vascular permeability, and thus greater susceptibility to capillary leak syndrome, in females than males. [6,35] We noted a difference in the effect of initial hemodynamic status by gender in the analysis of recurrent shock and profound DSS. Potentially, the higher intrinsic permeability in female subjects does influence the severity of leakage. However, further investigations are required to confirm and explain this observation.

Our prediction model provides a useful tool to predict development of profound shock among DSS cases, using basic clinical information gathered at onset of shock. The candidate predictors and primary outcome were prospectively defined, and the model was carefully developed and validated following standard methodology expected to minimize optimistic and/or spurious results. [21,23] The simplified model showed acceptable calibration and discrimination, with an AUC of 0.69 in internal validation, and performed favourably compared to a number of alternative modelling strategies. In temporal validation, all statistical models tended to overestimate the average risk of profound DSS in patients recruited in 2009 by about 5%, compared to models developed on patients recruited earlier during the study-period. We found no systematic linear time-trends in the risk for profound DSS over time and the same recruitment protocol and treatment regime were used throughout the study. However, the observed risk in 2009 was the lowest over the entire observation period (Fig 2). Although the over-estimation in 2009 could be a chance finding it is also possible that some undefined change did occur, only becoming apparent in 2009, but if so, the overall effect was minor.

We chose to simplify the final model for profound DSS to a score chart. While this approach results in a mild loss of precision, [21] a chart is easier to understand than a regression formula or nomogram, and allows clinicians to rapidly assess a patient’s risk of progression. In settings where smartphones are widely used by physicians, an alternative would be to implement a mobile application of the final model. We elected not to provide a clear-cut risk stratification or decision rule based on our prognostic model, since such a rule would require careful evaluation of costs and benefits, together with a defined intervention strategy for high-risk patients. Ideally all DSS cases should be managed in a high-dependency unit (HDU) or ICU, but such facilities are limited, especially in dengue-endemic areas, and given the very large numbers of potentially severe cases encountered daily, it can be difficult to prioritise individual cases. The contribution of our prognostic model is to provide physicians with guidance on the likely risk for developing profound shock Using this score physicians may elect to monitor high-risk patients closely in a local HDU setting, or may choose to transfer them early to tertiary-level facilities, allowing more effective use of available staff and equipment for the remaining DSS cases. In the wider context, a prediction model such as this could also be useful for identification of target populations for studies evaluating novel interventions for DSS. [38]

Although both models were based on Vietnamese children with DSS, our model differs from the only other published prediction model for recurrent shock in several respects. [15] We used data from 1207 patients from a single hospital, whereas in Huy’s study data of 444 patients from two very different health care settings were combined (a preventive health-care centre of a small province and a large referral hospital in a big city) although without reporting site-specific summaries. It is difficult to compare the models, as the reported proportions of patients with purpura/ecchymosis (36% vs. 3%) and ascites/pleural effusion (44% vs. 1%) were markedly different. Of note all data in our study were collected within 2 hours of onset of shock, while the timing of data collection in Huy’s study was not clearly specified; the high incidence of features that typically develop after initiation of fluid resuscitation suggests that timing of data collection may be relevant, and may explain why application of Huy’s model to our dataset showed only low discrimination (AUC 0.54) for the prediction of recurrent shock.

One potential limitation of our study relates to the definitions of the clinical outcomes. In the context of DSS where prompt diagnosis with immediate fluid resuscitation is very effective, [39] the occurrence of hard outcomes such as death depends heavily on local expertise and the facilities of the healthcare system. [40] As a specialist hospital with a very low mortality rate we elected to use outcomes based on specific therapeutic interventions that from the cornerstone of HTD’s DSS management protocol. Although care for sick ICU patients is invariably individualized to some extent, the hospital’s use of such guidelines follows the long-established precedent of protocol-driven therapy for DSS started by WHO more than 50 years ago; [39,41] adherence to guidelines is typically very good in Vietnam, and the senior PICU staff responsible for implementation remained unchanged throughout the study period. We believe that our definition of profound DSS is a robust assessment of the overall severity of DSS in these children, but physicians applying these results must understand our reasoning as well as the potential pitfalls inherent in this type of analysis.

Developing a prognostic model using data from a single hospital with better expertise and resources than many local healthcare facilities may be considered another limitation of the study, potentially restricting generalizability outside the primary context. However, as all the risk factors we identified are clinically plausible and results were highly consistent across endpoints, we anticipate that the model would also discriminate effectively in other settings. To adjust the model to provincial hospitals in Vietnam, where the distribution of predictors would likely be very similar to this study, simple re-calibration of the intercept of our prognostic model to take into account differences in outcome prevalence may be sufficient, and such a re-calibration could be performed with a much smaller sample size. [21] However, re-calibration or re-estimation of the regression coefficients would likely be required to adapt our model to settings with markedly different patient characteristics, facilities or management guidelines. [21] Further work is needed to assess the performance of the model in a variety of hospitals and clinics within the region, as well as more broadly across healthcare systems in parts of the world where dengue infection is less common. We believe this score-chart, simple to understand and easy to apply, could play a valuable role in triage and management of children with DSS in endemic areas, although precise prediction alone cannot improve clinical decision-making or overall outcomes.

## Supporting Information

S1 File Additional methods and details of the prognostic model development. (DOC)

S2 File Observational data of 1207 children with dengue shock syndrome in the cohort. (ZIP)
