# Supplementary material for: A Prognostic Model for Development of Profound Shock among Children Presenting with Dengue Shock Syndrome
Source: PLoS One. 2015 May 6;10(5):e0126134. doi: 10.1371/journal.pone.0126134 (PMC4422752; doi:10.1371/journal.pone.0126134)
Supplement: S1 File — (DOC) [file pone.0126134.s001.doc]

# Additional methods and details of the prognostic model development

# Study profile

## Table A:

**The study profile.**

| **Candidate predictors (assessed at enrolment)** | **Note** | | **Coding** |
| --- | --- | --- | --- |
| Age | Year | | continuous |
| Gender | Female / Male | | binary |
| Weight | kg | | continuous |
| Day of illness | Day of illness at shock | | continuous |
| Pulse rate | Beats per minute (“fast and weak” pulse was assigned as 200 beats/min) | | continuous |
| Temperature | Body temperature [°C] measured in the axilla | | continuous |
| Systolic blood pressure (SBP)^a^ | mmHg | | continuous |
| Pulse pressure (PP)^a^ | Difference between systolic and diastolic blood pressure (DBP) [mmHg], or assigned as 5mmHg if SBP measurable but DBP unmeasurable | | continuous |
| Hemodynamic Index^a^ | 1 if SBP ≥ lower limit of normal^b^ AND PP ≥ 10mmHg | | categorical |
|  | 2 if SBP < lower limit of normal OR PP<10mmHg | |  |
|  | 3 if SBP was unmeasurable | |  |
| Hemorrhage | ‘None’ if no bleeding at enrolment | | categorical |
|  | ‘Skin only’ if only petechiae/bruising at enrolment | |  |
|  | ‘Mucosal’ if epistaxis/gum/gastrointestinal/vaginal bleeding at enrolment | |  |
| Abdominal tenderness | Yes / No | | binary |
| Liver size | Size of liver below costal margin [cm] | | continuous |
| Haematocrit (HCT) | Haematocrit value [%] | | continuous |
| Platelet (PLT) | Platelet count [cells per mm3] | | continuous |
| **Patients** | **n** | **Note** | |
| Assessed for eligibility | 1298 |  | |
| Excluded | 91 | 82 did not have laboratory confirmation of dengue | |
|  |  | 6 had incomplete information on fluid usage | |
|  |  | 3 had severe bleeding within 12 hours of onset of shock (prior to achieving the criteria for profound shock based on vascular leakage) | |
| Primary analysis population | 1207 |  | |
| With profound shock (primary outcome) | 222 |  | |
| With recurrent shock (secondary outcome) | 433 |  | |

^a^ - Only hemodynamic index was included in the multivariable analysis

^b^ - Lower limit of normal SBP = 80mmHg (if age <5 years old) or 90mmHg (if age ≥ 5 years old)

# Assessment of models assumptions

The full logistic regression model assumes that all continuous candidate predictors affect the outcome linearly and additively, i.e. that there are no interactions. We assessed the validity of the linearity assumption numerically using likelihood ratio tests comparing the base model to models which included the selected continuous covariates in a more flexible way (i.e. either with an additional quadratic term or as a restricted cubic spline function with 4 knots). Graphically, we examined plots showing the estimated adjusted association of covariates with outcome based on a generalized additive model (GAM) with integrated smoothness estimation of continuous terms [1]. In addition, the additivity assumption of the model was assessed based on likelihood ratio tests for interactions between age, day of illness and hemodynamic index with all other covariates, respectively.

As shown in Table B, spline functions showed a significant improvement for modelling the effect of the day of illness at shock and HCT on the primary outcome of profound DSS while linear terms seemed to be adequate for other covariates. Consistent with these findings, plots showing the estimated adjusted association of covariates with outcome from a generalized additive model (GAM) also indicated potential non-linearity in the relationships between day of illness at shock and HCT with severity of disease (Fig A). However, these non-linear associations were driven by rare patients with highly unusual covariate values. Indeed, for the day of illness at shock, the increase of severity from day 7 onwards just represents the high proportion of profound DSS amongst 4 unusual patients who had shock later than day 7 (2 out of 4 had profound DSS). Similarly, for HCT, the high proportion of profound DSS amongst 5 patients who had HCT less than 40% (3 out of 5 cases had profound DSS) may distort the relationship between HCT and severity. Of note, 2 out of these 4 profound DSS cases had received intravenous fluid before enrolment into the study. The plots from a GAM-fit without these 9 unusual cases estimated a linear association with outcome, i.e. confirmed the adequacy of the simple model (Fig B). Because the day of illness at shock and HCT values of the unusual cases appeared correct and plausible, they were not excluded from the analysis even though these cases are not typical for dengue shock patients. However, due to their low number, they do not provide convincing evidence for a non-linear association.

The assessment of pre-defined interaction terms revealed a significant interaction between hemodynamic index and sex on both outcomes (Table C). Hemodynamic index is a categorical variable created by categorizing and combining systolic blood pressure and pulse pressure. To exclude that the interaction is an artefact from categorization in the definition of hemodynamic index categories, we fitted alternative models for the primary outcome of profound DSS which included systolic BP and pulse pressure as continuous covariates. This revealed that the interaction between hemodynamic index and sex can be explained by the interaction between systolic BP and sex and that the interaction remained when systolic BP was modelled as a continuous variable (p values of interaction tests are p=0.05 for all study participants and p=0.02 for patients with measurable systolic BP only, respectively).

## Table B:

**Summary of linearity tests in the pre-defined multivariable model for recurrent shock, profound DSS (N = 1207).**

| **Predictor** | **Non-linear transformation** | **df** | **Χ^2^ (p value)** | |
| --- | --- | --- | --- | --- |
|  |  |  | **Profound DSS** | **Recurrent shock** |
| Age | Quadratic | 1 | 1.40 (0.24) | 0.02 (0.89) |
|  | Restricted cubic spline with 4 knots | 3 | 6.83 (0.08) | 6.30 (0.10) |
| Weight | Quadratic | 1 | <0.01 (0.98) | 0.16 (0.69) |
|  | Restricted cubic spline with 4 knots | 3 | 4.36 (0.22) | 5.28 (0.15) |
| Day of illness | Quadratic | 1 | 2.51 (0.11) | 0.10 (0.75) |
|  | Restricted cubic spline with 4 knots | 3 | 8.46 (0.04) | 1.94 (0.58) |
| Pulse rate | Quadratic | 1 | 0.44 (0.51) | 6.37 (0.01) |
|  | Restricted cubic spline with 4 knots | 3 | 1.29 (0.73) | 7.29 (0.06) |
| Temperature | Quadratic | 1 | 0.57 (0.45) | <0.01 (0.98) |
|  | Restricted cubic spline with 4 knots | 3 | 2.83 (0.42) | 1.00 (0.80) |
| Haematocrit | Quadratic | 1 | 4.35 (0.04) | 0.99 (0.32) |
|  | Restricted cubic spline with 4 knots | 3 | 7.99 (0.05) | 3.27 (0.35) |
| Platelet count | Quadratic | 1 | 0.13 (0.72) | 0.32 (0.57) |
|  | Restricted cubic spline with 4 knots | 3 | 0.41 (0.94) | 0.80 (0.85) |

## Table C:

Interaction tests between selected variables and other covariates in the pre-defined multivariable model for recurrent shock and profound DSS (N = 1207).

| **Covariate** | **df** | **Χ^2^ (p value)** | |
| --- | --- | --- | --- |
|  |  | **Profound DSS** | **Recurrent shock** |
| Age and all other covariates^a^ | 14 | 16.7 (0.27) | 17.3 (0.24) |
| Day of illness at shock and all other covariates^a^ | 14 | 22.2 (0.07) | 11.0 (0.69) |
| Hemodynamic index and all other covariates^a^ | 24 | 35.6 (0.06) | 37.9 (0.04) |
| Hemodynamic index and sex | 2 | 9.9 (0.01) | 10.4 (0.01) |

^a^ - Overall likelihood-ratio test for interaction

## Fig A:

Plots of estimated component smooth functions of a generalized addictive model (GAM) fit for profound DSS with continuous covariates modelled using natural cubic spline functions and integrated smoothness estimation. Dots correspond to individual partial residuals; solid lines correspond to spline functions estimated by GAM; gray areas correspond to point-wise 95% confidence intervals of the estimated values.


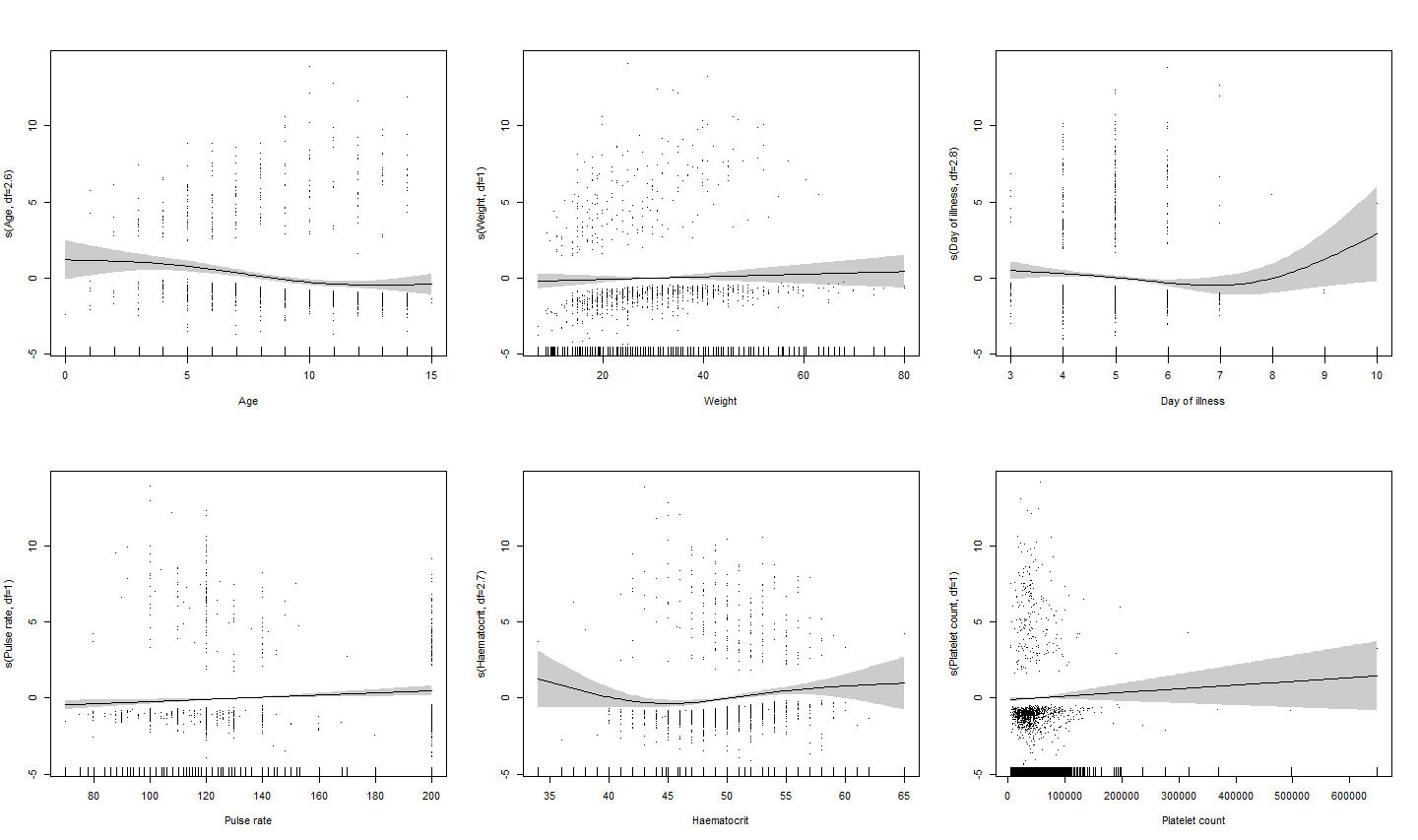


## Fig B:

Plots of estimated component smooth functions for day of illness and haematocrit from a generalized addictive model (GAM) fit for profound DSS after removal of 9 patients with day of illness > 7 or haematocrit values < 40%. Dots correspond to individual partial residuals; solid lines correspond to spline functions estimated by GAM; gray areas correspond to point-wise 95% confidence intervals of the estimated values.

**
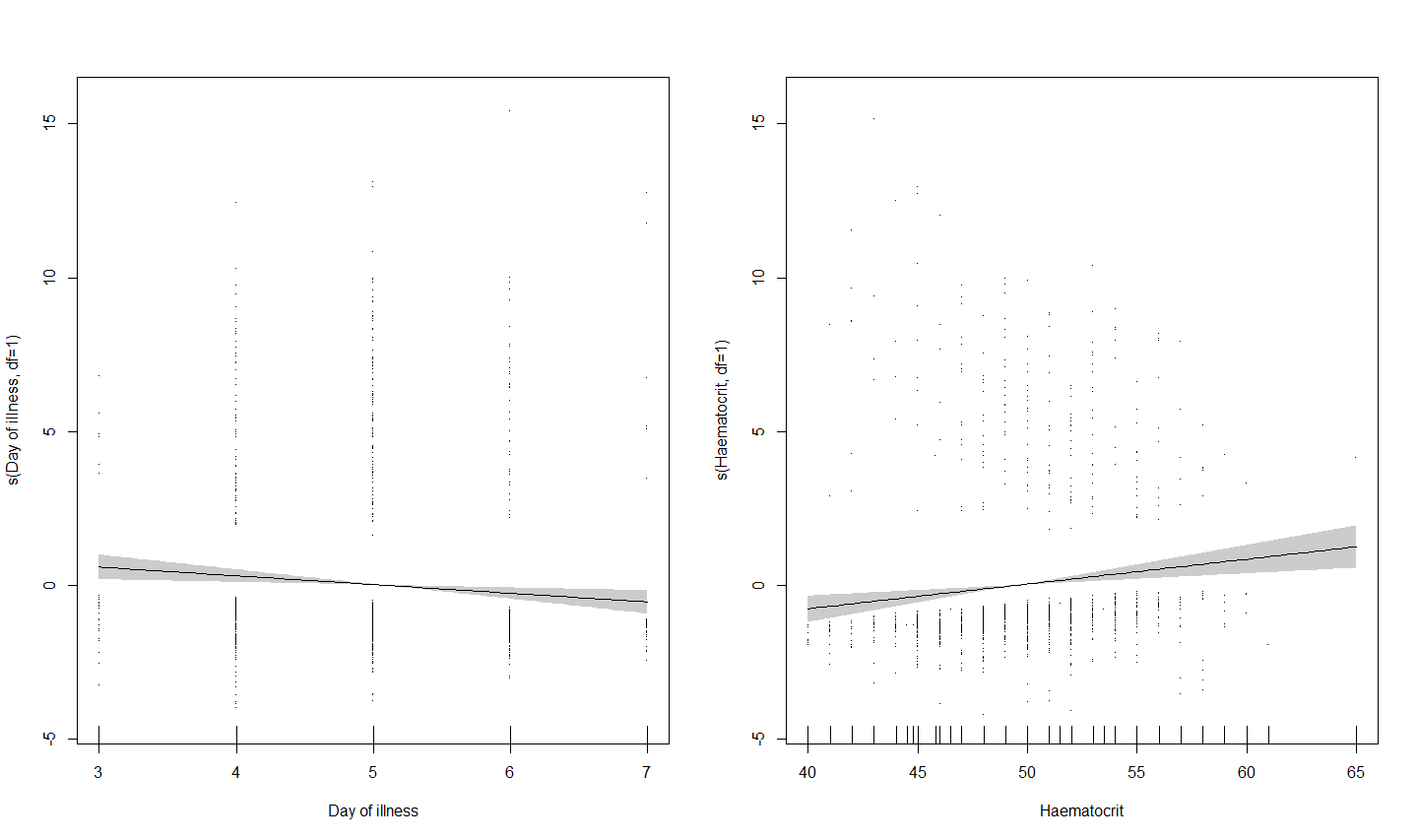
**

# Univariate analysis

## Table D:

Univariate effects of candidate predictors on clinical outcomes (N = 1207).

| **Candidate predictors** | **Profound DSS** | | |  | **Recurrent shock** | | |
| --- | --- | --- | --- | --- | --- | --- | --- |
|  | **OR** | **(95%CI)** | **p value** |  | **OR** | **(95%CI)** | **p value** |
| Age [+1 year] | 0.85 | (0.81, 0.89) | <0.01 |  | 0.89 | (0.86, 0.93) | <0.01 |
| Gender: male | 0.74 | (0.55, 0.99) | 0.04 |  | 1.02 | (0.81, 1.30) | 0.85 |
| Weight [+5 kgs] | 0.84 | (0.78, 0.90) | <0.01 |  | 0.89 | (0.84, 0.94) | <0.01 |
| Day of illness [+1 day] | 0.73 | (0.62, 0.87) | <0.01 |  | 0.77 | (0.67, 0.88) | <0.01 |
| Temperature [+1 ^0^C] | 1.47 | (1.07, 1.99) | 0.02 |  | 1.74 | (1.33, 2.31) | <0.01 |
| Pulse rate [+10 beats/min] | 1.14 | (1.10, 1.19) | <0.01 |  | 1.10 | (1.06, 1.13) | <0.01 |
| Systolic blood pressure [+ 5 mmHg] | 0.92 | (0.90, 0.94) | <0.01 |  | 0.96 | (0.94, 0.98) | <0.01 |
| Pulse pressure [+ 5 mmHg] | 0.61 | (0.55, 0.68) | <0.01 |  | 0.79 | (0.71, 0.87) | <0.01 |
| Hemodynamic Index |  |  | <0.01 |  |  |  | <0.01 |
| Group 1 | 1.00 |  |  |  | 1.00 |  |  |
| Group 2 | 1.67 | (1.19, 2.32) |  |  | 1.28 | (0.97, 1.68) |  |
| Group 3 | 5.06 | (3.11, 8.23) |  |  | 2.11 | (1.32, 3.37) |  |
| Hemorrhage |  |  | 0.43 |  |  |  | 0.55 |
| None | 1.00 |  |  |  | 1.00 |  |  |
| Skin only | 0.82 | (0.60, 1.12) |  |  | 0.88 | (0.68, 1.13) |  |
| Mucosal | 0.98 | (0.41, 2.11) |  |  | 0.79 | (0.38, 1.55) |  |
| Abdominal tenderness | 0.85 | (0.63, 1.15) | 0.29 |  | 0.99 | (0.78, 1.27) | 0.95 |
| Liver size [+1 cm] | 1.04 | (0.89, 1.21) | 0.65 |  | 1.11 | (0.98, 1.26) | 0.10 |
| Haematocrit [+1 %] | 1.07 | (1.03, 1.11) | <0.01 |  | 1.06 | (1.02, 1.09) | <0.01 |
| Platelet count [+ 10,000 cells/mm^3^] | 1.03 | (1.00, 1.07) | 0.07 |  | 1.01 | (0.98, 1.04) | 0.58 |
| AST [per 2-fold increase] ^a^ | 1.25 | (1.07, 1.45) | <0.01 |  | 1.37 | (1.20, 1.56) | <0.01 |
| Serotype ^a^ |  |  | <0.01 |  |  |  | 0.01 |
| DENV-1 | 1.00 |  |  |  | 1.00 |  |  |
| DENV-2 | 1.15 | (0.81, 1.61) |  |  | 1.26 | (0.95, 1.68) |  |
| DENV-3 | 2.46 | (0.90, 6.25) |  |  | 3.17 | (1.26, 8.62) |  |
| DENV-4 | 3.17 | (0.62, 14.54) |  |  | 1.39 | (0.27, 6.34) |  |
| Mixed | 0.60 | (0.03, 3.43) |  |  | 0.62 | (0.09, 2.70) |  |
| Negative | 0.51 | (0.31, 0.82) |  |  | 0.71 | (0.50, 1.01) |  |

**Footnotes for Table D**

DSS: dengue shock syndrome

OR: odds ratio

CI: confidence interval

AST: aspartate aminotransferase

^a^ AST and dengue serotype were included in the univariate analysis for descriptive purposes but not included in the prognostic model development as they are not usually available to clinicians at first presentation

# Validation of model development procedure

The whole model development process, except for the assessment of linearity and interactions, was temporally and internally validated. Temporal validation used data from the 939 patients enrolled before 2009 to develop a prognostic model and validated it on the 268 patients enrolled during 2009. Internal validation with 10-times 10-fold cross-validation repeatedly used nine tenths of the data for model development and one tenth for validation. [2,3]

# Interpretation of performance measures

Important aspects of model performance are discrimination, and calibration [3]. *Discrimination* measures how well a prognostic model can differentiate subjects with and without outcome. We assessed this aspect of model performance using the c-statistic defined as the area under the ROC curve (AUC). An AUC of 1 indicates perfect discrimination whereas an AUC of 0.5 indicates that the model does not discriminate better than random guessing [3].

*Calibration* measures the agreement between observed and predicted outcomes. We quantified calibration in terms of calibration-in-the-large and the calibration slope. Calibration-in-the-large assesses how well the average predicted risk matches the overall observed incidence of the outcome. The optimal value of calibration-in-the-large is 0. Calibration-in-the-large of <0 or >0, respectively, indicate that predicted outcomes are systematically too high or too low. The calibration slope reflects the extremeness of the predicted outcome and is compared to 1. A calibration slope < 1 indicates that the predictions are too extreme; whereas a calibration slope > 1 implies that the predictions are not extreme enough [3].

# Details regarding the comparison of the logistic model to alternative statistical approaches for the prediction of profound DSS

Estimation of the penalty parameter for the adaptive lasso was based on standardized covariates, estimates from the full logistic regression model as penalty weights, and 10-folds cross validation with the deviance as the optimization criterion as implemented in the R package glmnet version 1.9-8. The CART model built and pruned back a classification tree using default parameter settings of the R package rpart version 4.1-8. The GAM model was built based on default settings of the R package mgcv version 1.8-3. The implementation automatically estimates the degrees of freedom of smooth terms based on generalized cross-validation. To fit a “pure” additive model, the interaction term between sex and hemodynamic index was not included in the model formula. Finally, a generalized boosted regression model with a Bernoulli distribution for the outcome was fitted using classification trees as base learners as implemented in the R package gbm version 2.1-06. Each tree has a depth of at most 2 which allows for 2-way interactions. The number of 3000 iterations and the learning rate of 0.001 were chosen as recommended by the gbm package author.

None of the alternative models performed better than the primary logistic model with an interaction and AIC-based model selection (Table E). While several alternative models showed satisfactory performance, CART showed quite poor results.

## Table E:

Performance of alternative models for profound DSS based on internal and temporal validation (N = 1207).

| **Measure** | **Logistic 1** | **Logistic 2** | **Logistic 3** | **Logistic 4** | **Adaptive Lasso** | **CART** | **GAM** | **Boosting** |
| --- | --- | --- | --- | --- | --- | --- | --- | --- |
| **Internal validation** |  |  |  |  |  |  |  |  |
| AUC | 0.68 | 0.69 | 0.69 | 0.67 | 0.69 | 0.61 | 0.68 | 0.70 |
| Calibration-in-the-large | -0.02 | -0.02 | -0.02 | -0.02 | -0.02 | -0.02 | -0.01 | -0.03 |
| Calibration slope | 0.89 | 0.87 | 0.92 | 0.89 | 0.92 | 0.36 | 0.79 | 1.17 |
| **Temporal validation** |  |  |  |  |  |  |  |  |
| AUC | 0.71 | 0.73 | 0.74 | 0.68 | 0.73 | 0.61 | 0.69 | 0.72 |
| Calibration-in-the-large | -0.46 | -0.49 | -0.50 | -0.54 | -0.45 | -0.60 | -0.47 | -0.44 |
| Calibration slope | 1.06 | 1.13 | 1.22 | 0.99 | 1.27 | 0.37 | 0.83 | 1.21 |

Logistic 1: full logistic model without the interaction term between hemodynamic index and gender
Logistic 2: full logistic model with interaction (full model in Table 2 of the main text)
Logistic 3: logistic model with interaction and subsequent stepwise backwards model selection based on AIC (reduced model in Table 2 of the main text)
Logistic 4: logistic model with interaction and subsequent stepwise backwards model selection based on BIC

All performance measures in internal validation were corrected for optimism using 10-times 10-fold cross-validations.

# Derivation of the score chart

The logistic model with the most favourable trade-off between performance and simplicity was chosen as the basis for a score chart following the approach of Sullivan et al.[4]. In brief, we rounded and simplified the linear predictor of the selected model, followed by a categorization of continuous variables and assignment of a point value to each category of a covariate. The total point score for each patient obtained from the score chart is an approximation of the linear predictor corresponding to that patient which can then be converted to a predicted risk. Finally, the adequacy of this score chart was evaluated by comparing risk predictions from the score chart to those of the original logistic model, and by visualizing their agreement with a Bland – Altman plot [5].

The base model was the reduced logistic model (stepwise model selection using AIC) for profound DSS displayed in Table 2 of the main paper. However, results in Table 2 of the main manuscript give lower risk predictions in males with hemodynamic index group 2 compared to group 1. As the corresponding difference in estimates is small, non-significant, and clinically implausible, we pooled hemodynamic index groups 1 and 2 in males, and then refitted the logistic model prior to deriving the point score shown in Fig 2 of the main manuscript.

Evaluated based on all patients in this study, the score chart had a very similar apparent AUC as the logistic model with AIC model selection (AUCs were 0.72 and 0.71 for the logistic regression model and the score chart, respectively) (Fig C). We also assessed the adequacy of this score chart by comparing risk predictions from the score chart to those from the logistic model. The median (IQR) of the differences between these two risk-estimation approaches was 0.018 (-0.004, 0.035) and the range was -0.093 to 0.126. The largest differences occurred in patients with intermediate predicted risks (Fig D).

## Fig C:

ROC curves of the score chart and the logistic model for profound DSS with Akaike Information Criterion (AIC) model selection (and without pooling the hemodynamic index categories in males) based on the full data set (n=1207).


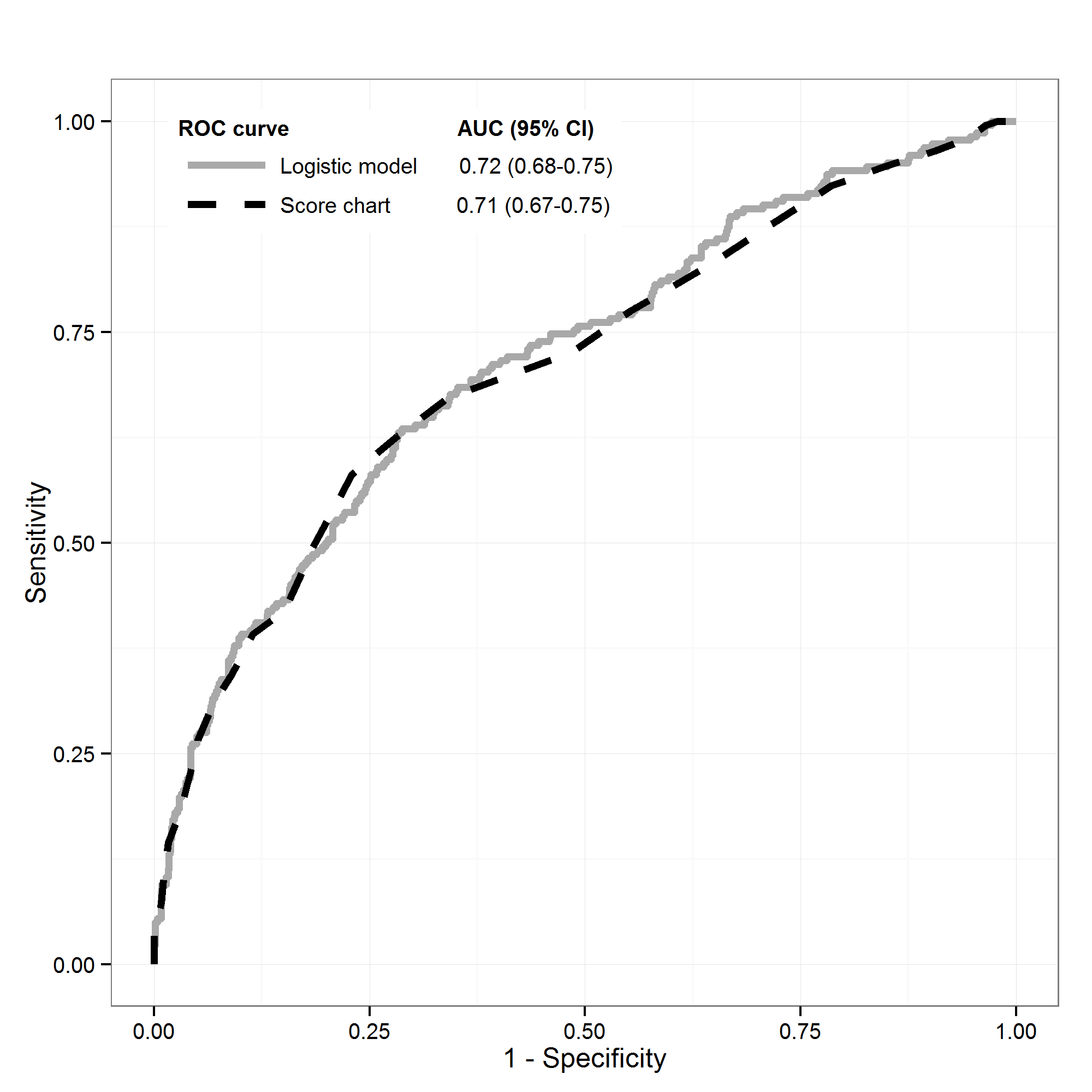


## Fig D:

Bland-Altman plot of differences between risks estimated by the score chart and those estimated by the logistic model for profound DSS with Akaike Information Criterion (AIC) model selection (and without pooling the hemodynamic index categories in males).

**
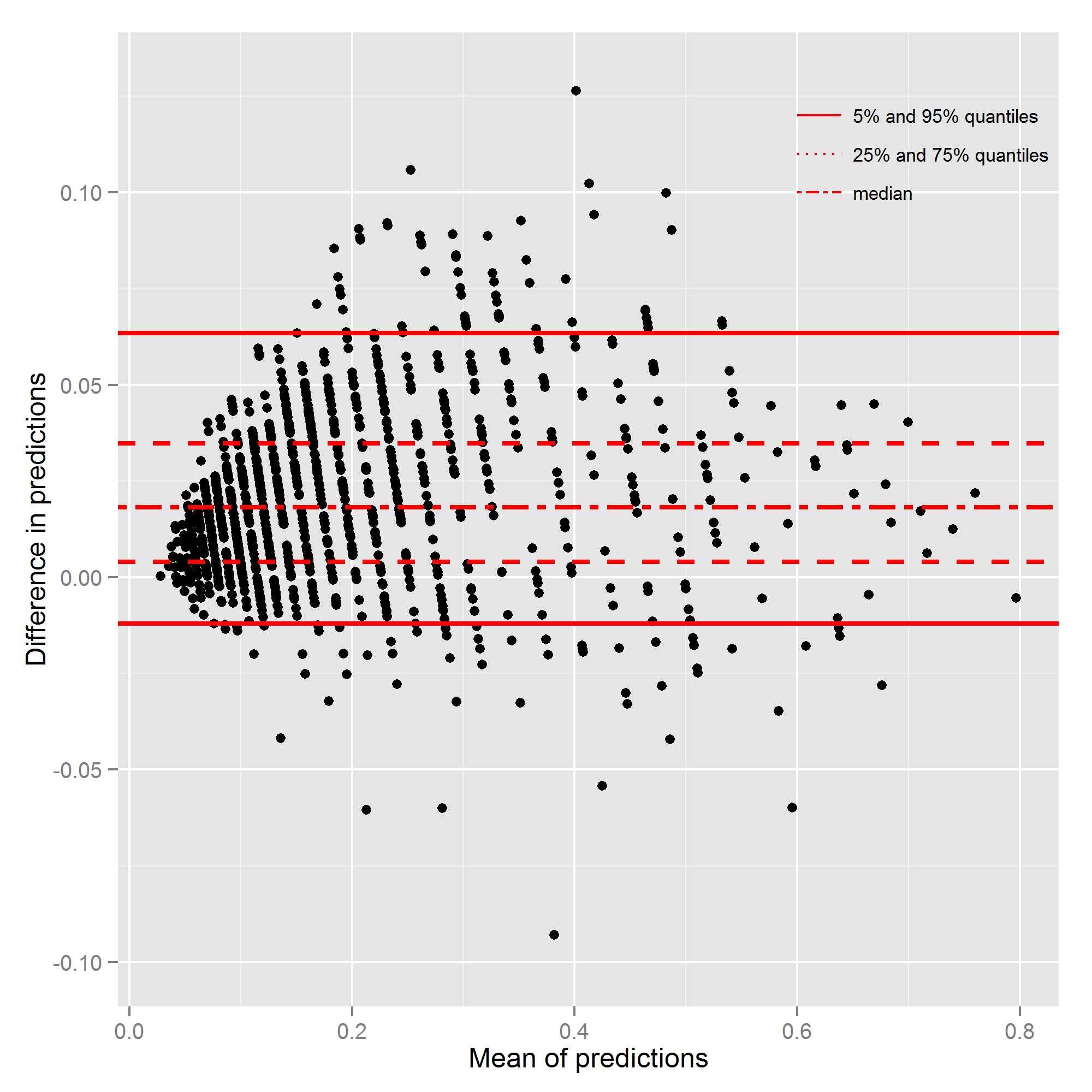
**

## References

1. Wood S (2006) Generalized Additive Models: An Introduction with R. 1st ed. Chapman and Hall/CRC.

2. Harrell FE (2001) Regression Modeling Strategies: with applications to linear models, logistic regression, and survival analysis. New York: Springer-Verlag.

3. Steyerberg EW (2010) Clinical Prediction Models: a practical approach to development, validation, and updating. New York: Springer.

4. Sullivan LM, Massaro JM, D’Agostino RB (2004) Presentation of multivariate data for clinical use: The Framingham Study risk score functions. Stat Med 23: 1631–1660.

5. Bland JM, Altman DG (1986) Statistical methods for assessing agreement between two methods of clinical measurement. Lancet 1: 307–310.
